# Neurophobia-Related Perceptions, Career Interest, and Educational Preferences Among Medical Students and Fresh Graduates at the University of Gezira, Sudan: A Cross-Sectional Study

**DOI:** 10.7759/cureus.114705

**Published:** 2026-08-18

**Authors:** Mahmoud H S. Daoud, Mohmed Hussien Ahmed Mohmed, Mohammed Muatasim Abbas Eltoom, Mustafa Osman, Maab Abdelmoniem Elhadi, Ebaa Elsir Massoud Mohamed, Mona Mahir Abdallah, Amna Taha Abdelwhab, Israa Mamoun, Eyas Mukhtar Abdelfetah Yousif, Ahmed Elawad

**Affiliations:** 1 Internal Medicine, University of Gezira, Wad Madani, SDN; 2 Neurology, Hatta Hospital, Hatta, ARE; 3 Medicine, Faculty of Medicine, University of Gezira, Wad Madani, SDN; 4 Anatomy, Omdurman Islamic University, Omdurman, SDN; 5 Internal Medicine, One Brooklyn Health - Interfaith Medical Center, New York, USA; 6 Pediatrics, Hatta Hospital, Hatta, ARE

**Keywords:** career interest, curriculum enhancements, neurology, neurophobia, sudan, university of gezira

## Abstract

Background: Neurophobia refers to perceived difficulty, fear of, and aversion to neurology and is a significant barrier in medical education globally, particularly in sub-Saharan Africa, where neurologists are scarce and disease burden is high.

Methods: A descriptive, cross-sectional study was conducted at the Faculty of Medicine, University of Gezira (FMUG), between December 2023 and March 2024. A self-administered questionnaire adapted from the previously validated NeuroQ questionnaire was distributed to 265 participants (medical students from Batches 40 and 41 and fresh graduates from Batch 39) using stratified random sampling. Descriptive statistics and chi-square tests were performed using the Statistical Package for the Social Sciences (SPSS); a p-value <0.05 was considered statistically significant.

Results: Of 265 participants (mean age: 24 ± 1 years; 171 (64.5%) female), 116 (43.7%) reported difficulty understanding neurological concepts, and 170 (64.2%) perceived neurology as more complicated than other specialties; over half (137, 51.6%) had difficulty applying theoretical knowledge clinically. The most common contributing factors were the influence of senior colleagues portraying neurology as difficult (182, 68.7%), neuroanatomy complexity (172, 64.9%), and short course duration (132, 49.8%). Only 51 (19.2%) expressed definite interest in a neurology career, while 127 (47.9%) were undecided. Problem-based learning (214, 80.7%) and bedside teaching (208, 78.5%) were the most preferred educational methods. Confidence in understanding neurological concepts was significantly associated with interest in pursuing neurology as a future career (χ² = 17.947, df = 8, p = 0.022).

Conclusion: Neurophobia-related perceptions were common among medical students and fresh graduates at the University of Gezira, particularly perceived difficulty in understanding neurological concepts, the belief that neurology is more complicated than other specialties, and difficulty applying theoretical knowledge in clinical settings, requiring curriculum enhancements and expanded bedside teaching.

## Introduction

Neurophobia, a term coined by Jozefowicz in 1994, describes the fear of and aversion to neurology among medical students and practicing physicians [1]. It encompasses perceived difficulty understanding neurological concepts, inadequate confidence in the clinical neurological examination, and reluctance to pursue neurology as a career [2]. Several factors have consistently been identified as drivers of neurophobia. Across diverse settings, including the United Kingdom [3], China [4], Brazil [5], and Lithuania [6], neuroanatomy is considered the primary academic hurdle because of its perceived complexity. The main problem is the disconnect between preclinical neuroscience education and clinical application, in which students learn the facts of neurology but find it difficult to apply them in practice [4,7].

Neurological diseases have a significant and growing global burden. Stroke, epilepsy, dementia, Parkinson's disease, multiple sclerosis, and other neurological disorders are among the leading causes of death and disability globally, accounting for nearly 10% of disability-adjusted life years (DALYs) [8]. In sub-Saharan Africa, this burden is disproportionately high, compounded by critical shortages of trained neurologists, limited access to neuroimaging, and underfunded neurological services [9]. With less than 0.03 neurologists per 100,000 people in many African countries, the region faces an urgent need to attract and train the next generation of neurologists [8,9].

Neurophobia is still widespread throughout the world despite this urgent need. A pooled neurophobia prevalence of 46% (95% confidence interval (CI): 35-57%) was found in a 2024 systematic review and meta-analysis of 24 studies from 30 countries (n = 10,395) [10]. However, a later meta-analysis of 32 studies found a pooled prevalence of 47.2% (95% CI: 39.8-54.6%), with medical students bearing a greater burden (52.3%) than residents and practicing physicians (41.9%) [11]. A cross-sectional survey of trainees in 15 African countries revealed that neurology was the most challenging medical subspecialty, and about one-third of them supported neurophobia [9].

Neurophobia has been associated with reduced interest in neurology as a career, threatening the global neurological workforce, particularly in low- and middle-income countries with limited treatment options, poor disease outcomes, and scarcity of neurologist role models [9]. Therefore, addressing neurophobia has important implications for both medical education and healthcare workforce development.

The Faculty of Medicine at the University of Gezira (FMUG) is recognized for its socially accountable and community-oriented approach to medical education and is a founding member of the Training for Health Equity Network [12]. Although it employs an innovative teaching approach, there has been limited research on neurophobia in this setting and across Sudan generally. It is crucial and timely to examine perceptions of neurophobia and their underlying factors at FMUG, given the substantial burden of neurological disorders in sub-Saharan Africa and the persistent shortage of neurologists and neurology training resources in the region [8,9].

The purpose of this study was to assess neurophobia-related perceptions among medical students and fresh graduates at FMUG, identify factors contributing to perceived difficulty in neurology, assess career interest in neurology, and identify preferred educational interventions.

## Materials and methods

Study design

This cross-sectional study used descriptive and analytical methods to assess neurophobia-related perceptions, career interest, and educational preferences among medical students and fresh graduates at FMUG, Sudan. Data were collected from medical students and fresh graduates using a modified questionnaire adapted from the previously validated NeuroQ questionnaire. The questionnaire comprised two sections: demographic data, including age, gender, and academic year; and data related to neurophobia, covering the concept of neurophobia, factors contributing to difficulty, career interest, discouraging and encouraging factors, preferred educational interventions, and perspectives toward neurophobia.

Study area and duration

The study was conducted at FMUG, Wad Medani, Gezira State, Sudan. Established in 1975, FMUG is a pioneer in community-oriented, problem-based medical education in Sudan. At FMUG, medical students are organized into batches by year of enrollment in a five-year (10-semester) undergraduate Bachelor of Medicine, Bachelor of Surgery program, with approximately 230-270 students per batch. Data were collected between December 2023 and March 2024.

Study population

The study focused on fresh graduates from Batch 39 and medical students from Batches 40 and 41, all of whom completed the course "The Nervous System and Its Problems" between January 2020 and December 2023.

Questionnaire validation

A panel of expert neurologists and community medicine specialists from the Internal Medicine and Community Medicine departments at FMUG reviewed the questionnaire to ensure that it measured the intended constructs and aligned with the study objectives. The instrument was adapted from the previously validated NeuroQ questionnaire [13] and pilot-tested with 20 medical students and graduates at the University of Gezira to assess its clarity, comprehensibility, and feasibility before the main study. The questionnaire was considered appropriate and required no major modifications; therefore, pilot participants were excluded from the final analysis. Internal consistency measures, such as Cronbach's alpha, were not calculated because the questionnaire was not designed to generate a composite neurophobia score. Instead, the items assessed distinct constructs, including perceived difficulty in neurology, confidence in understanding neurological concepts, and the ability to apply theoretical knowledge in clinical settings.

Eligibility criteria

Participants were eligible for inclusion if they were medical students from Batches 40 or 41 or fresh graduates from Batch 39 at the University of Gezira, all of whom had completed the undergraduate course "The Nervous System and Its Problems." Participants were required to provide informed consent before enrollment. Questionnaires were included in the final analysis only if at least 90% of items were completed and no core neurophobia assessment items were missing. Participants were excluded if they had not completed the aforementioned course, declined to participate, or submitted questionnaires with more than 10% missing responses or any omission from the core neurophobia assessment section.

Study variables

Outcome Variables

The study assessed individual neurophobia-related perceptions, including difficulty understanding neurological concepts and confidence in understanding neurological concepts. Career interest in neurology was also assessed as a separate categorical variable. These variables were analyzed individually rather than combined into a composite neurophobia score.

Explanatory Variables

Demographic and academic characteristics, including gender and educational level, were examined as explanatory variables in relation to selected neurophobia-related perceptions and career interest.

Measurement of Outcome Variables

Neurophobia-related perceptions were assessed using individual questionnaire items evaluating perceived difficulty in understanding neurological concepts and confidence in understanding neurological concepts. Career interest was assessed separately using the question, "Would you consider neurology as a future career?", with response options of Yes, No, and Maybe. Because the adapted questionnaire was not designed to generate a validated composite neurophobia score, these variables were analyzed individually.

Sample size and sampling technique

The sample size was calculated using the single population proportion formula, with a 95% confidence level, 5% margin of error, and an assumed prevalence of 50%, as no prior local study on neurophobia among medical students at the University of Gezira was available. Since the total source population was finite (N = 780), finite population correction was applied.

The minimum calculated sample size was 258 participants. To account for possible incomplete responses, additional questionnaires were distributed. A total of 265 complete responses were collected and included in the final analysis, comprising 83 fresh graduates from Batch 39 (out of 230), 93 students from Batch 40 (out of 280), and 89 students from Batch 41 (out of 270). A stratified random sampling technique was employed, whereby the study population was divided into three strata according to academic level (fresh graduates, Batch 40 students, and Batch 41 students), and participants were proportionally recruited from each stratum to ensure adequate representation.

Data collection procedure

Data were collected using a structured online self-administered questionnaire created and distributed through Google Forms (Google LLC, Mountain View, CA). Attitudinal items were measured using a five-point Likert scale: "Strongly disagree," "Disagree," "Neutral," "Agree," and "Strongly agree." Career interest in neurology was assessed separately using the question, "Would you consider neurology as a future career?" with three response options: "Yes," "No," and "Maybe." A written informed consent statement was included on the first page of the questionnaire, and only participants who provided electronic consent were able to proceed. Completed questionnaires were reviewed for completeness, consistency, and eligibility before inclusion in the statistical analysis.

Data entry and analysis

Data were entered, cleaned, and analyzed using the Statistical Product and Service Solutions (SPSS, version 23.0; IBM SPSS Statistics for Windows, Armonk, NY). Descriptive statistics, including frequencies, percentages, means, and standard deviations, were used to summarize participant characteristics and questionnaire responses.

For the Likert-scale items, responses were initially retained in their original five categories: "Strongly disagree," "Disagree," "Neutral", "Agree," and "Strongly agree." For the narrative presentation of results, "Strongly agree" and "Agree" responses were combined to represent overall agreement, while "Strongly disagree" and "Disagree" responses were combined to represent overall disagreement. Thus, percentages reported in the narrative for factors, perceptions, career-related factors, and preferred educational interventions represent the combined "Strongly agree + Agree" responses unless otherwise specified. The full five-category distributions were retained and presented in the corresponding tables.

Chi-square tests were used to explore associations between selected categorical variables. Specifically, associations were examined between educational level and difficulty understanding neurological concepts; educational level and career interest in neurology; gender and difficulty understanding neurological concepts; educational level and confidence in understanding neurological concepts; gender and career interest in neurology; and career interest in neurology and confidence in understanding neurological concepts. These analyses were exploratory and assessed relationships between neurophobia-related perceptions, demographic and academic characteristics, and career interest.

## Results

Participant characteristics

A total of 265 participants were included in the study. Females represented 171 (64.5%) of the sample, while males accounted for 94 (35.5%). The mean age was 24 ± 1 years (range: 20-33 years). Regarding educational status, 112 (42.3%) were students enrolled in the "Nervous System and Its Problems" course, 87 (32.8%) were clerkship students, and 66 (24.9%) were fresh graduates (Table 1).

**Table 1:** Demographic characteristics of the study participants (N = 265) SD = standard deviation. Age: mean ± SD = 24 ± 1 years; range: 20-33 years

Variable	N	%
Gender		
Male	94	35.5
Female	171	64.5
Educational level		
Completed Nervous System and Its Problems	112	42.3
Clerkship	87	32.8
Fresh graduates	66	24.9

Factors contributing to difficulty in the nervous system and its problems

The most frequently endorsed perceived source of difficulty in the "Nervous System and Its Problems" course was the influence of senior colleagues portraying neurology as difficult, with 182 (68.7%) participants selecting "Agree" or "Strongly agree." Studying neuroanatomy was the second most frequently endorsed perceived source of difficulty, with 172 (64.9%) participants selecting "Agree" or "Strongly agree," followed by the short duration of the course, endorsed by 132 (49.8%) participants (Table 2).

**Table 2:** Factors contributing to difficulty in the "Nervous System and Its Problems" (N = 265) SA = Strongly agree; A = Agree; N = Neutral; D = Disagree; SD = Strongly disagree; n = number of participants For narrative analysis, SA and A responses were combined to represent overall agreement, while D and SD responses were combined to represent overall disagreement.

Factor	SA n (%)	A n (%)	N n (%)	D n (%)	SD n (%)
Studying neuroanatomy is difficult.	56 (21.1)	116 (43.8)	33 (12.5)	43 (16.2)	17 (6.4)
Studying related neurosciences (neurophysiology and neuropathology) is difficult.	21 (7.9)	89 (33.6)	65 (24.5)	78 (29.4)	12 (4.5)
Taking a neurological history is difficult.	14 (5.3)	52 (19.6)	46 (17.4)	113 (42.6)	40 (15.1)
Performing a neurological clinical examination is difficult.	10 (3.8)	44 (16.6)	51 (19.2)	116 (43.8)	44 (16.6)
Understanding neurological diseases is difficult.	17 (6.4)	84 (31.7)	59 (22.3)	80 (30.2)	25 (9.4)
The duration of the "Nervous System and Its Problems" course is too short.	54 (20.4)	78 (29.4)	49 (18.5)	62 (23.4)	22 (8.3)
Senior colleagues’ perception that the "Nervous System and Its Problems" course is difficult and complicated contributes to my perception of difficulty.	97 (36.6)	85 (32.1)	37 (14.0)	31 (11.7)	15 (5.7)
Poor teaching methods make the "Nervous System and Its Problems" course difficult.	38 (14.3)	47 (17.7)	77 (29.1)	74 (27.9)	29 (10.9)

Neurophobia-related perceptions

Nearly half of the participants (116, 43.7%) reported difficulty understanding neurological concepts, while 170 (64.2%) perceived neurology as more complicated than other medical specialties. In addition, 137 (51.6%) reported difficulty applying theoretical neurological knowledge during clinical examination. Regarding self-confidence, 129 (48.7%) expressed confidence in understanding neurological concepts, and 136 (51.3%) felt confident in their ability to study neurology (Table 3).

**Table 3:** Neurophobia assessment statements (N = 265) SA = Strongly agree; A = Agree; N = Neutral; D = Disagree; SD = Strongly disagree; n = number of participants For narrative analysis, SA and A responses were combined to represent overall agreement, while D and SD responses were combined to represent overall disagreement.

Statement	SA n (%)	A n (%)	N n (%)	D n (%)	SD n (%)
I find neurological concepts difficult to understand.	38 (14.3)	78 (29.4)	67 (25.3)	52 (19.6)	30 (11.3)
Neurology is more complicated than other specialties.	59 (22.3)	111 (41.9)	47 (17.7)	31 (11.7)	17 (6.4)
I have confidence in understanding neurological concepts.	31 (11.7)	98 (37.0)	71 (26.8)	44 (16.6)	21 (7.9)
I have confidence in my ability to study neurology.	35 (13.2)	101 (38.1)	69 (26.0)	41 (15.5)	19 (7.2)
I find it difficult to apply theoretical knowledge to clinical examination.	42 (15.8)	95 (35.8)	64 (24.2)	42 (15.8)	22 (8.3)

Interest in neurology as a future career

When asked whether they would consider neurology as a future career, 51 (19.2%) participants responded "Yes," 87 (32.8%) responded "No," and 127 (47.9%) responded "Maybe." Among the 138 participants who expressed a definite position ("Yes" or "No"), 51 (37.0%) indicated an interest in pursuing neurology as a future career.

Factors discouraging a career in neurology

The main factors discouraging participants from choosing neurology were the difficulty of neuroanatomy and neuroscience, poor outcomes of neurological diseases, and limited treatment options (Table 4).

**Table 4:** Factors discouraging a career in neurology (N = 265) SA = Strongly agree; A = Agree; N = Neutral; D = Disagree; SD = Strongly disagree; n = number of participants For narrative analysis, SA and A responses were combined to represent overall agreement, while D and SD responses were combined to represent overall disagreement.

Factor	SA n (%)	A n (%)	N n (%)	D n (%)	SD n (%)
Neuroanatomy and basic neurosciences are tough and hard to study.	20 (7.5)	86 (32.5)	46 (17.4)	73 (27.5)	40 (15.1)
Clinical neurology is tough and hard to study.	13 (4.9)	46 (17.4)	64 (24.2)	91 (34.3)	51 (19.2)
Rare diseases, syndromes, and genetic disorders make neurology difficult.	22 (8.3)	76 (28.7)	66 (24.9)	74 (27.9)	27 (10.2)
Neurological examination is prolonged and difficult to perform.	13 (4.9)	51 (19.2)	59 (22.3)	84 (31.7)	58 (21.9)
Limited treatment options for neurological diseases.	35 (13.2)	78 (29.4)	73 (27.5)	52 (19.6)	27 (10.2)
Poor outcomes of neurological diseases.	47 (17.7)	70 (26.4)	63 (23.8)	45 (17.0)	40 (15.1)

Factors encouraging a career in neurology

The strongest motivating factor for pursuing neurology was the opportunity to make a difference in patients' lives (177, 66.8%). Other motivating factors included the challenging nature of neurological diagnosis, advances in neuroscience, and research opportunities (Table 5).

**Table 5:** Factors encouraging a career in neurology (N = 265) SA = Strongly agree; A = Agree; N = Neutral; D = Disagree; SD = Strongly disagree; n = number of participants For narrative analysis, SA and A responses were combined to represent overall agreement, while D and SD responses were combined to represent overall disagreement.

Factor	SA n (%)	A n (%)	N n (%)	D n (%)	SD n (%)
Financial reward	46 (17.4)	96 (36.2)	60 (22.6)	45 (17.0)	18 (6.8)
Ability to make a difference to patients’ lives	72 (27.2)	105 (39.6)	44 (16.6)	30 (11.3)	14 (5.3)
Research opportunities in neurology	76 (28.7)	91 (34.3)	63 (23.8)	22 (8.3)	13 (4.9)
Neurology is a rapidly advancing field.	66 (24.9)	103 (38.9)	61 (23.0)	23 (8.7)	12 (4.5)
Diagnosis of neurological conditions is challenging and stimulating.	66 (24.9)	105 (39.6)	53 (20.0)	30 (11.3)	11 (4.2)
Neurology is a prestigious career.	59 (22.3)	86 (32.5)	75 (28.3)	30 (11.3)	15 (5.7)
Good work-life balance in neurology	27 (10.2)	53 (20.0)	97 (36.6)	50 (18.9)	38 (14.3)

Preferred educational interventions

Problem-based learning and bedside teaching were the most preferred educational interventions to reduce neurophobia (Table 6).

**Table 6:** Preferred educational interventions (N = 265) PBL = problem-based learning; TBL = team-based learning; DR = discussion and review; SA = Strongly agree; A = Agree; N = Neutral; D = Disagree; SD = Strongly disagree; n = number of participants For narrative analysis, SA and A responses were combined to represent overall agreement, while D and SD responses were combined to represent overall disagreement.

Intervention	SA n (%)	A n (%)	N n (%)	D n (%)	SD n (%)
More problem-based learning (PBL)	96 (36.2)	118 (44.5)	28 (10.6)	19 (7.2)	4 (1.5)
More bedside teaching	88 (33.2)	120 (45.3)	40 (15.1)	13 (4.9)	4 (1.5)
More team-based learning (TBL)	87 (32.8)	97 (36.6)	42 (15.8)	24 (9.1)	15 (5.7)
More peer discussion	76 (28.7)	106 (40.0)	62 (23.4)	18 (6.8)	3 (1.1)
Increase duration of the course	89 (33.6)	86 (32.5)	47 (17.7)	28 (10.6)	15 (5.7)
More discussion and review (DR) sessions	73 (27.5)	102 (38.5)	51 (19.2)	26 (9.8)	13 (4.9)
More textbooks and study materials	44 (16.6)	122 (46.0)	53 (20.0)	36 (13.6)	10 (3.8)
More lectures	49 (18.5)	126 (47.5)	45 (17.0)	39 (14.7)	6 (2.3)
More online learning resources	32 (12.1)	97 (36.6)	49 (18.5)	60 (22.6)	27 (10.2)
More seminars	24 (9.1)	80 (30.2)	62 (23.4)	76 (28.7)	23 (8.7)

Associations between neurophobia-related perceptions, gender, educational level, and career interest

Additional analyses showed no significant associations between educational level and difficulty understanding neurological concepts (χ² = 3.174, df = 8, p = 0.923), educational level and interest in neurology as a future career (χ² = 0.414, df = 4, p = 0.981), gender and difficulty understanding neurological concepts (χ² = 4.939, df = 4, p = 0.294), or educational level and confidence in understanding neurological concepts (χ² = 3.774, df = 8, p = 0.877) and no significant gender difference in career interest in neurology (χ² = 4.640, df = 2, p = 0.098). However, a statistically significant association was observed between career interest in neurology and confidence in understanding neurological concepts (χ² = 17.947, df = 8, p = 0.022), with participants expressing interest in neurology reporting higher levels of confidence (Table 7).

**Table 7:** Association between confidence in understanding neurological concepts and interest in pursuing neurology as a future career

Career interest	Strongly agree n (%)	Agree n (%)	Neutral n (%)	Disagree n (%)	Strongly disagree n (%)	χ², df, p-value
No	24 (27.6)	24 (27.6)	35 (40.2)	3 (3.4)	1 (1.1)	χ² = 17.947, df = 8, p = 0.022
Yes	20 (39.2)	14 (27.5)	7 (13.7)	5 (9.8)	5 (9.8)	
Maybe	41 (32.3)	39 (30.7)	31 (24.4)	9 (7.1)	7 (5.5)	

## Discussion

Neurophobia, or a dislike of neurology among doctors and medical students [1], has a significant impact on neurological education, particularly in Sub-Saharan Africa [9]. This study demonstrated that neurophobia-related perceptions are common among medical students and fresh graduates at the University of Gezira. A substantial proportion of participants perceived neurology as more difficult than other medical specialties, reported challenges in understanding neurological concepts, and experienced difficulties applying theoretical knowledge in clinical practice. The most commonly identified contributors were the influence of senior colleagues, the complexity of neuroanatomy, and the short duration of neurology training.

In the current study, 170 (64.2%) of participants thought neurology was more difficult than other medical specialties, and 116 (43.7%) reported having trouble understanding neurological concepts. These findings are consistent with previous international and African studies, which have identified neurology as one of the most challenging medical disciplines and highlighted the widespread presence of neurophobia-related perceptions among medical trainees [9-11]. These findings suggest that Sudanese medical students and recent graduates experience challenges similar to those reported internationally.

About 137 (51.6%) of our participants reported difficulty applying theoretical knowledge of neurology during clinical examinations. This gap has been identified as a core driver of neurophobia, in which students acquire factual knowledge of the nervous system but struggle to translate it into clinical reasoning [7]. Han et al. similarly observed in a Chinese tertiary hospital survey that the inability to integrate preclinical and clinical neurological content was among the strongest predictors of neurophobia [4]. FMUG's current curriculum architecture may require structural enhancements to better strengthen integration of basic neuroscience with clinical neurology, given the persistent challenge across educational levels.

The most frequently endorsed contributing factor in our study was the influence of senior colleagues portraying neurology as difficult (182, 68.7%), followed by neuroanatomy complexity (172, 64.9%) and short course duration (132, 49.8%). Senior peer influence plays an especially important role, but it has received relatively little attention in the literature when compared to curriculum-level factors. According to the Neurosciences Journal, senior doctors may unintentionally pass on their own fear of neurology to junior trainees, perpetuating a neurophobia cycle across generations of physicians [14]. These findings suggest that interventions that enhance senior students' and postgraduate trainees' attitudes through mentorship training and role modeling may be as crucial as curriculum reform.

Neuroanatomy was the second most frequently endorsed perceived source of difficulty in the "Nervous System and Its Problems" course, with 172 (64.9%) participants agreeing or strongly agreeing that studying neuroanatomy contributed to their difficulty with the subject. This finding is consistent with previous studies that have identified neuroanatomy as a commonly perceived challenge in neurology education [3-6]. The perceived difficulty of neuroanatomy may reflect both the complexity of neuroanatomical concepts and challenges in integrating anatomical knowledge with clinical application [7]. At FMUG, the existing problem-based learning infrastructure could provide opportunities to integrate neuroanatomical concepts into clinically oriented scenarios, potentially improving their relevance and facilitating learning and retention. However, given the descriptive cross-sectional design of the present study, these findings should be interpreted as participants’ perceptions rather than evidence that neuroanatomy independently contributes to or causes neurophobia.

A total of 132 (49.8%) participants deemed the short duration of a neurology course as a contributing factor to neurophobia, aligning with findings from low- and middle-income countries, where limited exposure to neurology during training is a key modifiable structural issue [15]. Extending the duration of the Nervous System and Its Problems course or distributing neurology learning across multiple years of the curriculum could meaningfully address this deficiency.

Although many participants found neurology difficult, about half were confident in their understanding of neurological concepts and ability to study neurology. Similar findings were reported by Lithuanian medical students, who described neurology as difficult but interesting and well-taught [6]. Further longitudinal studies are needed to assess whether this confidence persists during clinical training and influences career choice.

A total of 51 (19.2%) participants expressed a definite interest in pursuing neurology, while 127 (47.9%) remained undecided. This finding is consistent with reports from the 15-country African survey by McDonough et al. (2022) [9]. A statistically significant association was observed between interest in neurology and confidence in understanding neurological concepts, with participants interested in neurology reporting greater confidence than those who were undecided or not interested (χ² = 17.947, df = 8, p = 0.022). This finding is consistent with AlZahrani et al. (2025), whose global systematic review identified perceived competence and confidence as factors associated with reduced neurophobia and greater interest in neurology [11]. The substantial proportion of undecided participants may represent an opportunity for targeted educational interventions, including mentorship, increased clinical exposure, and competency-based teaching, to strengthen confidence and support informed career decision-making regarding neurology.

Careers in neurology are significantly discouraged by worries about poor neurological disease outcomes and limited treatment options, particularly in sub-Saharan Africa, which has the lowest neurologist-to-population ratio and severely limited diagnostic and therapeutic resources [15]. Such perceptions may perpetuate a cycle in which a lack of interest in neurology contributes to a shortage of neurologists. To combat students' negative attitudes toward treatment outcomes, improvements in neurology education and investment in neurological care capacity are essential.

The primary motivations for pursuing a career in neurology are the desire to positively impact patients' lives (177, 66.8%), the intellectual challenges of neurological diagnosis (171, 64.5%), and the potential for neuroscience research (167, 63.0%). Students appeared intrinsically motivated but were hindered by perceived barriers. Therefore, highlighting these positive aspects of neurology may help improve perceptions of the specialty and increase interest among undecided students.

Regarding gender, some studies have identified female gender as an independent risk factor for neurophobia. Kam et al. found that female gender (odds ratio (OR): 3.0; 95% CI: 1.3-6.7) independently increased the risk of neurophobia among Singapore medical students [16]. Our findings indicate no significant gender difference in career interest in neurology (χ² = 4.640, df = 2, p = 0.098), despite a higher disinterest among females 63 of 171 (36.8%) than males (24 of 94 (25.5%). The predominance of female participants 171 (64.5%) may have restricted the statistical power to detect gender-based differences, suggesting that larger samples could yield significant results.

The study found that 214 (80.7%) and 208 (78.5%) of participants preferred problem-based learning and bedside teaching, respectively, as educational interventions, aligning with current research on neurophobia prevention strategies. A longitudinal study by Shiels et al. (2017) found that problem-based learning, team-based learning, and case-based teaching significantly enhanced students' self-reported neuroscience knowledge, with most valuing these modalities as beneficial [17]. A 2024 crossover trial found that the Analogy, Neural signs, Written practice, Engagement, and Recall (ANSWER) multimodal teaching intervention, combining analogy, clinical scenarios, sign enactment, and recall learning, significantly reduced neurophobia scores and improved medical students' knowledge [18]. FMUG, the largest teaching hospital outside Khartoum, should systematically leverage its existing infrastructure for expanded bedside neurology teaching.

Students strongly endorse extending the neurology course (175, 66.0%), providing more discussion sessions (175, 66.0%), and incorporating more peer discussion (182, 68.7%) to master clinical neurology. This result is consistent with suggestions that, to reduce neurophobia, neurological education should include a continuum of learning, active teaching strategies, and a more robust integration of fundamental neuroscience with clinical practice [19]. The faculty's educational philosophy would be naturally extended by incorporating these strategies into FMUG's problem-based, community-oriented model.

Given the growing burden of neurological disorders and the limited neurology workforce in Sudan and other low- and middle-income countries, addressing neurophobia should be considered an educational priority. Curriculum enhancements, integration of basic neuroscience with clinical practice, and increased clinical exposure may improve students' perceptions of neurology and encourage greater interest in the specialty.

Limitations

Several limitations of this study should be recognized. First, the cross-sectional design precludes causal inference regarding factors associated with neurophobia-related perceptions and does not allow assessment of their longitudinal trajectory across different stages of medical training. Second, the study was conducted at a single institution, which may limit the generalizability of the findings to other medical schools in Sudan or the region. However, FMUG admits students from different regions of Sudan, which may provide some diversity within the study population.

Third, although the questionnaire was adapted from NeuroQ [13], no validated composite neurophobia score was calculated; therefore, the study assessed neurophobia-related perceptions rather than estimating the prevalence of neurophobia itself. In addition, ranking perceived sources of difficulty according to the combined proportions of "Agree" and "Strongly agree" identifies the most frequently endorsed perceptions but does not establish that these factors independently contribute to or predict neurophobia. Fourth, the use of self-reported data may have introduced recall and social-desirability biases. Finally, the relatively narrow range of educational levels, all of which had completed the "Nervous System and Its Problems" course, may have limited the ability to detect differences in neurophobia-related perceptions across levels of training that might be observed in a broader cohort spanning preclinical students to senior postgraduate trainees.

## Conclusions

A substantial proportion of medical students and fresh graduates at the University of Gezira reported neurophobia-related perceptions, including difficulty understanding neurological concepts, the perception that neurology is more complicated than other medical specialties, and difficulty applying theoretical knowledge in clinical practice. Key contributing factors included the influence of senior colleagues, the perceived complexity of neuroanatomy, and the short duration of neurology training. Interest in pursuing neurology as a career was low, although many participants identified altruistic motivations as encouraging factors. Problem-based learning and bedside teaching were the most highly endorsed educational interventions and may represent valuable strategies for improving neurology education. These findings suggest that addressing neurophobia-related perceptions through curriculum enhancements and increased clinical exposure may help strengthen neurology training and support workforce development in regions facing a growing burden of neurological disease and a shortage of neurologists. Additionally, future multicenter studies using standardized neurophobia assessment tools are warranted to confirm these findings and evaluate the effectiveness of educational interventions.
